# Investigation of the efficacy of paclitaxel on some miRNAs profiles in breast cancer stem cells

**DOI:** 10.3906/biy-2103-46

**Published:** 2021-10-18

**Authors:** Elif ERTÜRK, Ferda ARI, Oğuzhan AKGÜN, Engin ULUKAYA, Cem İsmail KÜÇÜKALİ, Ümit ZEYBEK

**Affiliations:** 1 Vocational School of Health Services, Bursa Uludağ University, Bursa Turkey; 2 Department of Biology, Science and Art Faculty, Bursa Uludağ University, Bursa Turkey; 3 Department of Clinical Biochemistry, Faculty of Medicine, İstinye University, İstanbul Turkey; 4 Department of Neuroscience, Aziz Sancar Experimental Medicine Research Institute, İstanbul University, İstanbul Turkey; 5 Department of Molecular Medicine, Aziz Sancar Experimental Medicine Research Institute, İstanbul University, İstanbul Turkey

**Keywords:** Breast cancer,, stem cells,, MCF-7s,, miR-125b,, paclitaxel

## Abstract

Understanding of the functions of microRNAs in breast cancer and breast cancer stem cells have been a hope for the development of new molecular targeted therapies. Here, it is aimed to investigate the differences in the expression levels of let-7a, miR-10b, miR-21, miR-125b, miR-145, miR-155, miR-200c, miR-221, miR-222 and miR-335, which associated with gene and proteins in MCF-7 (parental) and MCF-7s (Mammosphere/stem cell-enriched population/CD44+/CD24-cells) cells treated with paclitaxel. MCF-7s were obtained from parental MCF-7 cells. Cytotoxic activity of paclitaxel was determined by ATP assay. Total RNA isolation and cDNA conversion were performed from the samples. Changes in expression levels of miRNAs were examined by RT-qPCR. Identified target genes and proteins of miRNAs were analyzed with RT-qPCR and western blot analysis, respectively. miR-125b was significantly expressed (2.0946-fold; p = 0.021) in MCF-7s cells compared to control after treatment with paclitaxel. Downregulation of SMO, STAT3, NANOG, OCT4, SOX2, ERBB2 and ERBB3 and upregulation of TP53 genes were significant after 48 h treatment in MCF-7s cells. Protein expressions of SOX2, OCT4, SMAD4, SOX2 and OCT4 also decreased. Paclitaxel induces miR-125b expression in MCF-7s cells. Upregulation of miR-125b may be used as a biomarker for the prediction of response to paclitaxel treatment in breast cancer.

## 1. Introduction

Breast cancer (BC) is the most common and deadly cancer type among women in the world. Despite improvements in BC therapy, local-regional recurrence and distant metastasis still continues (Guo et al., 2019). Cancer stem cells (CSCs) are shown as one of the main reasons why today’s treatments are not provided with sufficient effectiveness. CSCs are a small cell population that differs from less tumorigenic cancer cells that make up the overall tumor mass, with the ability to selfrenewal and differentiation to many different cells (Mertins, 2014; Phi et al., 2018). However, it is estimated that these cells are not only responsible for the formation of new tumors but also for the resistance to recurrence and chemotherapy (Ari et al., 2013; Aztopal et al., 2018; Mertins, 2014; Phi et al., 2018). Studies in recent years support this hypothesis and reveal that there are many factors in inducing the differentiation of CSCs (Aztopal et al., 2018; Mertins, 2014; Phi et al., 2018).

MicroRNAs (miRNAs) are small single-stranded RNA molecules, approximately 18–24 nucleotides in length, encoded from highly conserved DNA regions but not translated into protein (Ling et al., 2013). These protein-encoding RNA molecules bind to target mRNAs that are complementary to their nucleotide sequences and perform posttranscriptional gene expression regulation by translational suppression or mRNA degradation (Flatmark et al., 2016). Thus, miRNAs are involved in many metabolic pathways to provide hemostasis in the cell. They are especially effective in processes such as cell growth, differentiation, cell death mechanisms and apoptosis. In many studies, it has been stated that miRNAs affect metabolic processes in many cancers including BC (Ling et al., 2013; Bertoli et al., 2016; Flatmark et al., 2016).

The effectiveness of cancer treatment is often limited to acquired resistance to chemotherapy. Despite progress in identifying molecular determinants of the cancer chemotherapy response, a comprehensive understanding of the mechanisms underlying drug resistance is still a challenge (Mansoori et al., 2017). Paclitaxel is a type of taxane antimicrotubule drug. It usually acts on the G2-M phase (inhibits mitosis) and leads to apoptotic cell death. However, it controls intercellular signaling by regulating gene expression levels. This compound is commonly utilized in BC chemotherapy due to its unique role in stabilizing microtubule polymerization and polymerized microtubules. At the present time, although paclitaxel is involved in first-line therapy, the molecular mechanisms of paclitaxel therapy are still unknown (N. Chen et al., 2014; Samli et al., 2019). Recently, changes in miRNA expression profiles have revealed that BC is highly associated with function of CSCs and cancer therapy resistance (Mansoori et al., 2017; Loh et al., 2019). To date, studies on miRNA expression alterations in BC have been widely performed in cell lines and clinical samples, but the number of studies performed in CSCs is very few (Bertoli et al., 2015; Prabhu et al., 2020).

Therefore, our study aims to investigate differences in expression levels on MCF-7 and MCF-7s cells of 10 of miRNAs (let-7a, miR-10b, miR-21, miR-125b, miR-145, miR-155, miR-200c, miR-221, miR-222 and miR-335) that were previously shown to be related to BC prognosis, drug response and CSC biology. We have found that miR-125b seems to be a candidate biomarker that deserves further attention. 

## 2. Materials and methods

### 2.1. Cell lines and chemicals

BC cell line, MCF-7, was cultured in RPMI 1640 Medium (Gibco, USA) supplemented with100 U/mL penicillin (+) 100 μg/mL streptomycin (Gibco, USA), 2 mML-glutamine (Gibco, USA), and 5% fetal bovine serum (Gibco, USA), at 37 °C in a humidified atmosphere containing 5% CO_2_. Paclitaxel (2 mg/mL) was obtained from the pharmacy of the Medical School of Bursa Uludağ University. Paclitaxel was aliquoted and stored at room temperature and diluted in culture medium.

### 2.2. Mammosphere (stem cell-enriched population) culture from MCF-7 cell line

After centrifugation, the MCF-7 cell pellet was prepared as a single cell suspension and seeded (2.5×10^5^ cells/mL) in 25 cm^2^ ultralow attachment cell culture flasks (Corning Inc., Corning, NY) at 37 °C in a humidified atmosphere containing 5% CO_2_. The protocol in the previous study was followed for the mammosphere culture medium (Aztopal et al., 2018).Mammosphere (stem cell-enriched population)isolated by magnetic separation and then measured CD44+/CD24- percentage, which breast cancer stem cells marker by flow cytometry as given Supplementary Figure.After the mammosphere structures were formed (3–4 days), they were collected by 800G 10 min centrifugation and mechanically-enzymatically (Tryple Select; Gibco, USA) decomposed. Single cells were then replated for subsequent passages.

### 2.3. The ATP viability assay

Depending on the luminescence-based methodology, the ATP method can be performed much more sensitively and reliably than other viability methods in terms of in vitro cytotoxicity measurements. Cellular ATP is the most sensitive endpoint for measuring cell viability. ATP content that is perfectly correlated with viability, meaning the less ATP, the less viability, even meaning no ATP, no viability**.** The level of intracellular ATP content is an indicator used to determine the number of living cells (Andreotti et al., 1995; Dexter et al., 2003; Ulukaya et al., 2008). ATP cell viability assay was carried out following the manufacturer’s instructions (ATP Bioluminescent Assay Kit, Sigma, Steinheim, Germany). MCF-7s cells were seeded on 96-well ultralow attachment cell culture plate in triplicates at a density of 5×10^3 ^cells per well and treated with the drug. Paclitaxel (0.25–15.93 µM) was applied at different concentrations. Cells were incubated with the treatment groups for 24 and 48 h at 37 °C in a 5% CO_2_. After the treatment period, the ATP assay was performed as previously depicted (Ulukaya et al., 2008).

### 2.4. RNA isolation from parental MCF-7 and MCF-7s specimens

MCF-7 and MCF-7s cells were seeded at a density of 1×10^5^ cells per well of a 6-well plate and cells were harvested for RNA isolation 24 and 48h after the treatment with paclitaxel (3.98 µM). RNA isolation was performed accordance with the manufacturer’s instructions using the total RNA purification kit (Thermo Fisher Scientific, USA). RNA concentration was measured using NanoDrop 2000 (Thermo Fisher Scientific, USA).

### 2.5. Real-time quantitative PCR-based miRNA expressions

The 10 most important miRNAs (Table 1) in BC development (Calin et al., 2006; Iorio et al., 2008; Visone et al., 2009; Erturk et al., 2014) were analyzed in MCF-7 and MCF-7s cells. cDNA synthesis was performed with the SCRIPT cDNA synthesis kit (Thermo Fisher Scientific, USA) using 500 ng of total RNA for each group according to the manufacturer’s specifications. Then, MCF-7 and MCF-7s cells were analyzed on an Applied Biosystems Step One Plus Real-Time PCR (Thermo Fisher Scientific, USA). Analyses were made in triplicate for each sample and two independently experiment. To evaluate the miRNA expression, RNU6 was used as reference gene. The average Ct values of this housekeeping gene from this assay was used as a baseline to normalize the miRNA expression data and to increase the accuracy. 

**Table 1:** miRNAs in BC development.

Accesion ID	miRNA	Localization	Putative Function
MIMAT0000062	hsa-let-7a	22q13.31	TS
MIMAT0000254	hsa-miR-10b	2q31.1	O
MIMAT0000076	hsa-miR-21	17q23.2	O
MIMAT0000423	hsa-miR-125b	11q24.1	TS
MIMAT0000437	hsa-miR-145	5q32	TS
MIMAT0000646	hsa-miR-155	21p21.3	O
MIMAT0000617	hsa-miR-200c	12p13.31	TS
MIMAT0000278	hsa-miR-221	Xp11.3	O
MIMAT0000279	hsa-miR-222	Xp11.3	O
MIMAT0000765	hsa-miR-335	7q32.2	TS

TS: Tumor supressor, O: Oncogenic.

Relative expression was calculated using the comparative Ct method. The fold change of miRNA expressions was evaluated by the 2^–ΔCt^ method (Livak et al., 2001). A web-based software package was used for data analysis (http://pcrdataanalysis.sabiosciences.com/mirna/arrayanalysis.php).

### 2.6. Identification of differentially expressed miRNA target genes

In order to identify miRNA target genes, miRBase (http://www.mirbase.org) and Targetscan (http://www.targetscan.org) were identified by scanning. Furthermore, MiRTarBase and MiRDB were examined to evaluate the target genes of statistically significant miRNA. 

### 2.7. Evaluation of the expression level of miRNA target genes

To determine the expression of the target genes of miR-125b, RNAs were reverse transcribed using a cDNA synthesis kit (Thermo Fisher Scientific, USA). Next, expression changes of target genes were examined by RT-PCR using the samples obtained (Table 2). The expression level of the human glyceraldehyde 3-phosphate dehydrogenase (GAPDH) housekeeping gene was also evaluated. Gene expression analyses were made in triplicate for each sample and two independently experiment. The initial copy number of the samples and the threshold cycle (Ct) for mRNA expression were identified using the Step One Plus Real-Time PCR (Applied Biosystems, Thermo Fisher Scientific, USA). The 2^−ΔCt^ method was also used in calculations (Livak et al., 2001).

**Table 2:** Primer sets used for RT-qPCR.

Gene	Forward	Reverse
ERBB2	5’ ACC TGC TGA ACT GGT GTA TG 3’	5’ GAC TCT TGA CCA GCA CGT T 3’
ERBB3	5’ GAC ACA ATG CCG ACC TCT C 3’	5’ GTT GGG CAA TGG TAG AGT AGA G 3’
TP53	5’ TGG TTC TAT GAC TTT GCC TGA TAC 3’	5’ CAT TCA GCT CTC GGA ACA TCT C 3’
STAT3	5’ TAC AGT GAC AGC TTC CCA ATG 3’	5’ CAC CAA AGT GGC ATG TGA TTC 3’
SMO	5’ CAA GCT CGT GCT CTG GTC 3’	5’ ATT CTC ACA CTT GGG CAT GTA 3’
BCL2	5’ GTG GAT GAC TGA GTA CCT GAA C 3’	5’ GAG ACA GCC AGG AGA AAT CAA 3’
OCT4	5’ GGA GGA AGC TGA CAA CAA TGA 3’	5’ CTC TCA CTC GGT TCT CGA TAC T 3’
SOX2	5’ CAC CTA CAG CAT GTC CTA CTC 3’	5’ TGG GAG GAA GAG GTA ACC A 3’
NANOG	5’ TCC TGA ACC TCA GCT ACA AAC 3’	5’ GCG TCA CAC CAT TGC TAT TC 3’
PARP	5’ TGA CCA GCA GAA AGT CAA GAA 3’	5’ CAA AGT CAC CCA GAG TCT TCT C 3’
GAPDH	5’ AAC AGC CTC AAG ATC ATC AGC 3’	5’ GCG TCA AAG GTG GAG GAG TG 3’

### 2.8. Western blotting 

Western blot technique was conducted as described previously (Akgun et al., 2019). The membranes were probed sequentially with SMAD family member 4 (SMAD4) (#38454), sex determing region Y HMG-box 2 (SOX2) (#3579), octamer-binding transcription factor (OCT4) (#2750), epidermal growth factor receptor (EGFR) (#4267) and GAPDH (#2118) antibodies (Cell Signaling Technology, MA, USA). Then, HRP-linked anti-rabbit IgG antibodies (Cell Signaling Technologies, MA, USA) were used. HRP bound to membranes were visualized with the Fusion FX-7 chemiluminescence imaging system (Vilber Lourmat, Torcy, France). The experiment was repeated two independently experiment.

### 2.9. Statistical analysis

To examine the effect of miRNA expressions on MCF-7 and MCF-7s cells, a statistical analysis was performed. RT2 Profiler PCR Array Data Analysis (http://www.sabiosciences.com/pcr/arrayanalysis.php) Spss (v23) and GraphPad Prism 8 (Demo Version; GraphPad, San Diego, CA) were used to investigate whether miRNA expressions were significant on MCF-7 and MCF-7s. The significance was calculated using Independent Sample T Test and one-way analysis of variance (ANOVA). Confidence intervals of 95% were calculated using the associated estimated standard errors. A value of p < 0.05, p < 0.01, and p < 0.001 was considered statistically significant.

## 3. Results 

3.1. Cytotoxicity of paclitaxel on MCF-7s (stem cell-enriched population)

**Figure 1:**
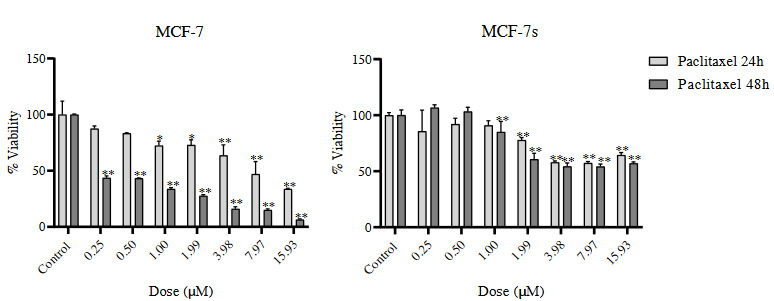
Viability of MCF-7 and MCF-7s cells after treatment with paclitaxel for 24 and 48 h measured by the ATP viability assay. *Denotes statistically significant differences in comparison with control: *(p < 0.01); **(p < 0.001). Data are presented as mean ± SD (n = 3).

**Figure 2:**
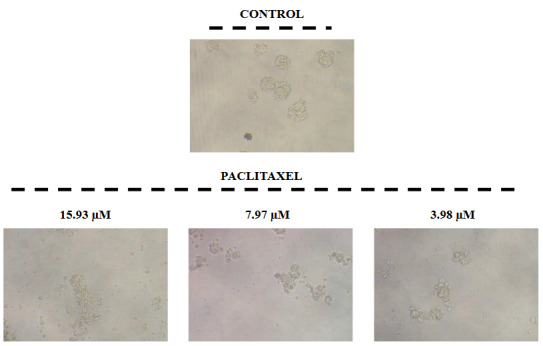
The effects of paclitaxel treatment on mamosphere formation for 48 h. (X20).

ATP viability assay was used to evaluate the cytotoxic effect of paclitaxel treatment in both MCF-7 and MCF-7s. Different concentrations of paclitaxel (0.25–15.93 µM) were used for 24 and 48 h (Figure 1). In MCF-7 and MCF-7s cells, paclitaxel treatment was determined to decrease cell viability depending on time and dose administered (Figure 1, p < 0.01, p < 0.001). When MCF-7s cell viability were evaluated, we observed that viability haven’t gone below 50%. IC_50_ (concentration that kills 50% of cells) values also support these results. This data shows that MCF-7s cells are more resistant to paclitaxel treatment compared to MCF-7 cells (Figure 1, Table 3). In addition, in microscopic images, we observed that sphere structures were disrupted after the addition of paclitaxel (Figure 2). 

**Table 3:** IC50 values during 24 and 48 h of treatment according to the results of ATP viability administered with paclitaxel.

Cell Line	IC50 (24 h)	IC50 (48 h)
MCF-7	7.26 μM	<0.25 μM
MCF-7s	>15.93 μM	>15.93 μM

### 3.2. Evaluation of the miRNA expressions after paclitaxel treatment in MCF-7s

An expression study of 10 miRNAs demonstrated that some of the miRNAs were expressed at different levels after treatment of paclitaxel (3.98 µM) compared with the control cells. Results revealed that, miR-155 was 2.0186-fold downregulated for 24 h and the expression of let-7a was 2.8154-fold (p = 0.000041), miR-10b was 3.5227-fold, miR-221 was 2.9828-fold and miR-335 was 3.1456-fold downregulated in MCF-7 cells for 48 h. However, 9 miRNAs (let-7a, miR-10b, miR-21, miR-125b, miR-145, miR-200c, miR-221, miR-222 and miR-335), (1.3629- to 4.9819-fold) and 6 miRNAs (miR-21, miR-125b, miR-145, miR-155, miR-200c and miR-222), (1.057- to 13.737-fold) were upregulated in MCF-7 cells (for 24 and 48 h, respectively). Among the miRNAs that upregulated in 48 h, the expression of let-7a was 2.835-fold (p = 0.010) and miR-21 was 2.5315-fold (p = 0.000096) remarkable upregulated (24 and 48 h, respectively), (Table 4). 

**Table 4:** Time dependent miRNA expressions in MCF-7 cells.

		let-7a	miR-155	miR-10b	miR-221	miR-222	miR-335	miR-145	miR-200c	miR-21	miR-125b
24 h	2^(-Avg.(Delta(Ct))	0.05954	0.004688	0.014714	0.011385	0.056328	0.020617	0.770215	0.069992	1.494849	1.561934
	Fold Change	2.835	0.4954	2.7511	4.9818	3.1675	3.6978	2.5609	1.3629	1.4845	2.1092
	Fold Regulation	2.835	–2.0186	2.7511	4.9818	3.1675	3.6978	2.5609	1.3629	1.4845	2.1092
	95% CI	(1.91–3.76)	(0.00001–1.09)	(0.00001–8.44)	(0.00001–13.41)	(0.80–5.54)	(0.00001–8.93)	(0.00001–5.58)	(0.20–2.52)	(0.80–2.17)	(0.00001–4.98)
	*p value	0.010262	0.356686	0.394606	0.218809	0.060603	0.19278	0.205594	0.40021	0.18924	0.311871
48 h	2^(–Avg.(Delta(Ct))	0.037421	0.040107	0.002619	0.004304	0.023089	0.023303	0.95705	0.112656	1.81085	4.521078
	Fold Change	0.3552	6.0349	0.2839	0.3353	4.1892	0.3179	13.737	1.5157	2.5315	1.057
	Fold Regulation	–2.8154	6.0349	–3.5227	–2.9828	4.1892	–3.1456	13.737	1.5157	2.5315	1.057
	95% CI	(0.31–0.40)	(0.00001–36.73)	(0.00001–1.11)	(0.00001–0.88)	(0.00001–12.99)	(0.00001–1.53)	(0.00001–43.68)	(0.14–2.89)	(2.25–2.82)	(0.30–1.82)
	*p value	0.000041	0.370039	0.354623	0.346856	0.306847	0.818284	0.053877	0.344629	0.000096	0.891101

*p values evaluated by independent sample T test with comparing control groups 2^(-Avg.(Delta(Ct)).

On the other hand, 6 miRNAs (miR-21, miR-125b, miR-155, miR-200c, miR-222 and miR-335) were downregulated 1.0093- to 28.443-fold and 4 miRNAs (let-7a, miR-10b, miR-145 and miR-221) were upregulated 1.0918- to 1.6283-fold in MCF-7s for 24 h. However, 3 miRNAs (let-7a, miR-155 and miR-221) were downregulated 1.1225- to 3.1456-fold and 7 miRNAs (miR-10b, miR-21, miR-125b, miR-145, miR-200c, miR-222 and miR-335) were upregulated 1.5511-to 27.0958-fold in MCF-7s for 48 h. Among these miRNAs, miR-125b was 2.094-fold significantly upregulated in MCF-7s compare to control group after 48 h treatment (p = 0.021), (Table 5). There was no change in miR-125b expression after 24 h of treatment.

**Table 5:** Time dependent miRNA expressions in MCF-7s cells.

		let-7a	miR-155	miR-10b	miR-221	miR-222	miR-335	miR-145	miR-200c	miR-21	miR-125b
24 h	2^(-Avg.(Delta(Ct))	0.248273	0.011951	0.01019	0.064854	0.036314	0.010648	1.107009	0.002421	0.882703	2.265768
	Fold Change	1.146	0.9117	1.107	1.6283	0.3711	0.1334	1.0918	0.0352	0.9908	0.1665
	Fold Regulation	1.146	–1.0968	1.107	1.6283	–2.6945	–7.4988	1.0918	–28.443	–1.0093	0.6071
	95% CI	(0.00001– 3.37)	(0.00001– 6.39)	(0.00001– 4.71)	(0.23– 3.03)	(0.00001– 1.41)	(0.00001– 0.58)	(0.00– 2.18)	(0.00001– 0.20)	(0.00001– 2.54)	(0.03–0.30)
	*p value	0.65884	0.378155	0.955554	0.420976	0.446397	0.345634	0.642654	0.401774	0.628202	0.36387
48 h	2^(–Avg.(Delta(Ct))	0.151075	0.001532	0.028099	0.002416	0.041426	0.129408	0.705475	0.027268	1.04006	7.81728
	Fold Change	0.8909	0.3798	4.8121	0.3179	1.6857	27.0958	10.2674	1.5511	3.1895	2.0946
	Fold Regulation	–1.1225	–2.6329	4.8121	–3.1456	1.6857	27.0958	10.2674	1.5511	3.1895	2.0946
	95% CI	(0.00001– 4.98)	(0.00001– 2.23)	(0.00001– 25.78)	(0.00001– 1.30)	(0.00001– 5.48)	(0.00001–152.76)	(0.00001– 41.99)	(0.00001– 4.99)	(0.00001– 7.67)	(1.02–3.17)
	*p value	0.428215	0.657663	0.820488	0.362657	0.483842	0.368128	0.330658	0.813157	0.213482	0.02145

*p values evaluated by independent sample T test with comparing control groups 2^(–Avg.(Delta(Ct)).

### 3.3. Effects of paclitaxel treatment on target genes of miR-125b in MCF-7s

**Figure 3:**
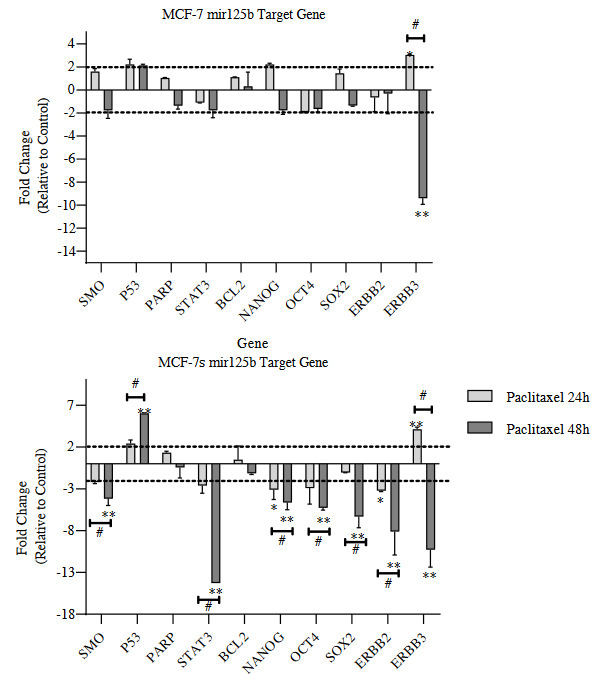
Gene’s expression levels in MCF-7 and MCF-7s cells. Cells were treated with paclitaxel (3.98 μM) 24 and 48 h, expression changes in target genes of miR-125b were quantified by RT‐qPCR. *Denotes statistically significant differences in comparison with control: *(p < 0.01), **(p < 0.001). #Denotes statistically significant differences between groups (Paclitaxel 24 h and 48 h):#(p < 0.001). Data are presented as mean ± SD (n = 3). GAPDH, glyceraldehyde 3-phosphate dehydrogenase; RTqPCR, quantitative real-time polymerase chain reaction; SD, standard deviation.

Upregulation of miR-125b was found statistically significant after 48 h of treatment in MCF-7s cells. For this reason, miR-125b was targeted in gene and protein analyzes. The target genes of miR-125b were determined by searching from the literature and online databases. Expression levels of these genes were evaluated by RT-qPCR (Banzhaf-Strathmann et al., 2014; Y. Wang et al., 2020). Among these selected genes OCT4, SOX2, Nanog Homeobox (NANOG), signal transducer and activator of transcription 3 (STAT3) and smoothened (SMO) are important in resistance to anti-cancer drugs, differentiation and self-renewal of CSCs (Ben-Porath et al., 2008; Lengerke et al., 2011; Zhao et al., 2011; Leis et al., 2012; Nagata et al., 2014; Galoczova et al., 2018); tumor protein p53 (TP53) and Poly (ADP-ribose) polymerase-1 (PARP) are effective in repair mechanisms, apoptosis and CSC biology (Zeniou et al., 2019; Uhlmann et al., 2020); B-Cell Leukemia/Lymphoma 2 (BCL2) is involved in apoptosis processes and CSC survival (Czerwinska et al., 2015) and finally Erb-B2 receptor tyrosine kinase 2 (ERBB2) and Erb-B3 receptor tyrosine kinase 3 (ERBB3) are responsible for determining the aggressive properties and drug resistance of BCs (J. Chen et al., 2018). In general, these genes were selected because they are associated with BC therapy resistance and CSC biology. RT-qPCR analysis demonstrated that expression levels of SMO, STAT3, NANOG, OCT4, SOX2, ERBB2 and ERBB3 genes were decreased in MCF-7s cells after 48 h treatment. In addition, TP53 gene expression level was increased on MCF-7s cells in 48 h (Figure 3). The differences in expression levels and the fold change values are given in Table 6. 

**Table 6:** Fold change of genes that showed differences in expression.

	Cell Line	SMO	TP53	PARP	STAT3	BCL2	NANOG	OCT4	SOX2	ERBB2	ERBB3
24 h	MCF–7	1.60333	2.21667	1.06333	–1.08773	1.12000	2.22000	–1.91190	1.45000	–0.64461	3.05000
48 h		–1.75519	2.14667	–1.35088	–1.75171	0.33101	–1.76012	–1.63528	–1.33587	–0.31186	–9.39394
24 h	MCF-7s	–2.18496	2.43667	1.33333	–2.60860	0.49288	–3.10994	–2.90650	–1.03853	–3.22805	4.12000
48 h		–4.16667	6.02667	–0.44856	–14.28571	–1.14284	–4.64905	–5.27290	–6.31410	–8.11966	–10.27778

The reduction in expression levels of these genes (SMO, STAT3, NANOG, OCT4 and SOX2) that are effective in CSCs regulation has shown that paclitaxel suppresses the stem cell properties of MCF-7s cells and the treatment is effective. In addition, after 48 h of treatment, paclitaxel has also been demonstrated to trigger downregulation of ERBB2 and ERBB3, which are associated with poor prognosis. Another finding that supports the efficacy of the treatment is the triggering the induction of apoptosis with an increase in TP53 expression level.

### 3.4. Effects of paclitaxel treatment on target proteins of miR-125b in MCF-7s 

**Figure 4:**
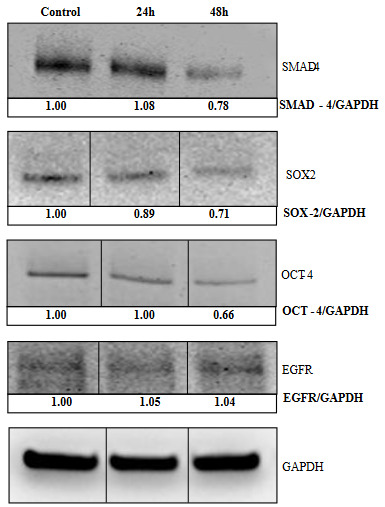
Protein expression changes in MCF-7s cells. MCF-7s were treated with paclitaxel (3.98 μM) 24 and 48 h, SMAD4, SOX2, OCT4 and EGFR protein expression changes quantified by western blotting. Densitometry was performed with the ImageJ software and densitometric analysis of the observed bands’ intensity normalized to GAPDH and quantified with respect to controls set to 1.0. GAPDH, glyceraldehyde 3-phosphate dehydrogenase. Data are presented as mean ± SD (n = 3).

To detect the protein expression levels of SMAD4, SOX2, OCT4, and EGFR that were potential targets of miR-125b, we also performed western blotting (Lengerke et al., 2011; Zhao et al., 2011; Leis et al., 2012; Masuda et al., 2012; Liu et al., 2014). Among these SMAD4 is a downstream mediator of transforming growth factor beta (TGF-β) that regulates cell proliferation of CSCs, differentiation, apoptosis and cancer progression (Liu et al., 2014). EGFR (also known as ErbB1 and HER1), ERBB2 (HER2/neu and HER2), ERBB3 (HER3), and ERBB4 (HER4), members of the (EGFR)/(ERBB) family, are known among the most important cancer molecular targets. EGFR overexpression is related with therapy resistance, cell proliferation, angiogenesis and CSCs enrichment in BC (Masuda et al., 2012; Czerwinska et al., 2015; J. Chen et al., 2018). As with gene selection, these proteins were also evaluated for their suitability to our study and were preferred because of their role in signal pathways, which are important in BC development, drug resistance and stem cell regulation. Western blot analysis of the present study indicated that SMAD4, SOX2 and OCT4 protein expressions were downregulated in paclitaxel treatment after 48 h (Figure 4).

This result showed that after 48 h treatment with paclitaxel in MCF-7s cells caused a decrease in stem cell markers (SMAD4, SOX2, and OCT4). However, the expression of EGFR after 24 and 48 h of treatment did not change significantly. In addition, there was no significant change after 24 h treatment compared to control.

## 4. Discussion

The incidence of BC ranks first among women in the world and second in overall rankings. It accounts for 25% of all new cancer cases (Bray et al., 2018). Despite the decline in mortality rates due to advances in early diagnosis and treatment, BC is still responsible for the reason of death in millions of women in the world. Although screening methods are effective for the detection of BC at the treatable stage, current methods are not sufficient for all BCs (Erturk et al., 2014). For this reason, new prognostic and diagnostic biomarkers are needed in BC to achieve better disease outcome and prolong patient survival. As a result of the determination that miRNAs act as regulators of gene expression, these molecules have become both possible therapeutic targets and identified as candidate indicators for diagnosis and prognosis (Erturk et al., 2014; Bertoli et al., 2015; Adhami et al., 2018).

In the current study, differences in the expression levels of 10 miRNAs effective in BC prognosis (let-7a, miR-21, miR-125b, miR-145, miR-155, miR-200c), drug response (let-7a, miR-21, miR-125b, miR-221, miR-222) and CSC biology (let-7a, miR-10b, miR-21, miR-125b, miR-155, miR-221, miR-222, miR-335) were evaluated in the parental MCF-7 and MCF-7 (stem cell-enriched population) after 24 and 48 h of treatment with paclitaxel. Our findings showed that although there was no change in miR-125b expression level after 24 h treatment, the expression level increased 2.091–fold after 48 h of paclitaxel treatment in MCF-7s cells compared to control cells (p = 0.021). miR-125b is related with invasion and metastasis, drug response and stem cell regulation in different types of cancer, including BC (Emmrich et al., 2014; Vilquin et al., 2015). Although miR-125b plays an important regulatory role in BC, two distinct roles of this miRNA have been reported (Nie et al., 2019). Some studies demonstrated that breast tumor tissues express low of miR-125b and that it plays a role in tumor suppression (Banzhaf-Strathmann et al., 2014; Vilquin et al., 2015; Y. Wang et al., 2020). However, other researchers noticed that miR-125b overexpression in BC cells stimulated paclitaxel resistance and high miR-125b expression level was a biomarker for poor prognosis in BC (Vilquin et al., 2015; Zhou et al., 2015). These different results might be associated with tissue or cell specific effects, recommending that different miRNA expression in humans could detect functional specificity between tissue and cell and might include complex gene regulation (Banzhaf-Strathmann et al., 2014; Nie et al., 2019; Y. Wang et al., 2020). Our findings showed that miR-125b increased 2.094-fold after 48 h of treatment. This revealed that miR-125b functions as a tumor suppressor and increases the efficacy of paclitaxel treatment. 

Chemotherapy resistance in BC is one of the main obstacles for treatment and one of the distinguishing features of BCSCs. Studies have revealed the potential role of miR-125b on chemoresistance by regulating BCSC (N. Chen et al., 2014; J. Chen et al., 2018; Y. Wang et al., 2020). Wang et al. (2013) analyzed the function of miR‐125b in BC stem cell‐like side population. And they found that overexpression of miR‐125b correlated with an increase in stem cell‐like tumor side population and CSC property. Also, miR-125b showed positive correlation with paclitaxel in chemoresistance (S. Wang et al., 2013). Furthermore, Vilquin et al.’s (2015) in their work found that ectopic overexpression of miR-125b or miR-205 and silencing of miR-424 expression in sensitive MCF-7aro cells, play a role in gaining resistance against letrozole and anastrozole. They detected that upregulation of miR-125b expression was associated with poor prognosis in BC (Vilquin et al., 2015). Moreover, Kong et al. (2011) reported that the upregulation of miR-125b in ovarian cancer is associated with cisplatin resistance (Kong et al., 2011).

The roles of miR-125b have not been fully elucidated in different tumor types, including breast tumors. Although the studies in the literature emphasize that the increase of miR-125b expression contributes to paclitaxel resistance, in our study, the effectiveness of paclitaxel treatment might be increased as a result of the increase in miR-125b expression. In addition, the increase in miR-125b expression supported paclitaxel therapy. This result showed that the upregulation of miR-125b determined tumor suppressive properties and, thus, increased the effectiveness of paclitaxel treatment in this study. However, if this level of regulation differs during carcinogenesis, oncogenic or tumor suppressor pathways are activated or blocked. Therefore, we think that increased or decreased miR-125b expression in different tumors may contribute to carcinogenesis (Banzhaf-Strathmann et al., 2014; Y. Wang et al., 2020).

To understand the functional mechanism of miR-125b, it is critical to identify targets involved in their regulation. Through analysis using miRBase, TargetScan, MİRTarBase and MİRDB, a number of important candidate targets for miR-125b were predicted (Y. Wang et al., 2020). Among these potential targets, downregulation of SMO, STAT3, NANOG, OCT4, SOX2, ERBB2, ERBB3 and upregulation of TP53 were significant in MCF-7s cells after 48 h treatment. SOX2, OCT4, and NANOG are CSC markers associated with tumor proliferation and tumor differentiation (Ben-Porath et al., 2008; Lengerke et al., 2011; Zhao et al., 2011; Leis et al., 2012). In previous studies, in many carcinomas, including BC, high expression of these genes has been identified as a predictive biomarker for poor prognosis (Ben-Porath et al., 2008; Kong et al., 2011; Czerwinska et al., 2015). Also, it was determined that combinational high expression of OCT4, SOX2, NANOG and other transcription factors had roles in programming of somatic cells into pluripotent stem cell-like cell types (Ben-Porath et al., 2008; Lengerke et al., 2011; Zhao et al., 2011; Leis et al., 2012). In addition, Ben-Porath et al. (2008) reported in their study that SOX2, OCT4 and NANOG expressions were high in BCSCs. In the current study, downregulation of these CSC related markers after 48 h of treatment showed that paclitaxel treatment is effective. It can be thought that high miR-125b expression in MCF-7s cells indirectly suppresses the expression of these markers by supporting paclitaxel treatment. Besides other functions, the expression level of STAT3, which is effective in terms of its ability to promote cancer by regulating CSCs activities in tumor biology, with SMO, which is one of the components of the hedgehog (Hh) signaling pathway that plays a role in embryonic development, were also determined in low regulation (Galoczova et al., 2018). The reduction in expression levels of these genes that are effective in CSCs regulation has shown that after 48 h treatment, paclitaxel suppresses the stem cell properties of MCF-7s cells, and it was found to be successful for treatment. In addition, after 48 h of treatment in MCF-7s cells, paclitaxel has also been demonstrated to trigger downregulation of ERBB2 and ERBB3, which are associated with poor prognosis ( Masuda et al., 2012; J. Chen et al., 2018). Another finding showing the effectiveness of treatment is the increased in the expression level of TP53-induced apoptosis (Uhlmann et al., 2020). Previous studies demonstrated that these genes are essential for the regulator of cell proliferation and maintenance of CSCs (Ben-Porath et al., 2008; Czerwinska et al., 2015). Also, overexpression of these genes is associated with cancer development and poor prognosis. In the present study, the high expression level of miR-125b mediates downregulation of these genes, suppressing the efficacy of paclitaxel in MCF-7s cells after 48 h treatment.

For finding out the effect of miR-125b in protein expression of its targets, four important targets, SMAD4, SOX2, OCT4 and EGFR were selected from its validated targets (Y. Wang et al., 2020). As reported above, SOX2 and OCT4 are stem cell predictor factors that induce the expression of each other, regulate cancer progression, and are biomarkers of CSCs (Ben-Porath et al., 2008). On the other hand, serving as the central mediator of TGF-β signaling pathway, SMAD4 is a potential prognostic marker of breast carcinoma development, and it is critical for stem cells’ self-renewal (Liu et al., 2014). Thus, it was determined that the expression levels of CSCs markers decreased in both gene and protein analyzes. However, other potential mechanisms still need to be explored to develop new therapeutic methods.

## 5. Conclusion

In summary, it seems that miR-125b upregulation may be a predictive biomarker for effect of paclitaxel on CSCs of BC. In addition, paclitaxel has a satisfactory potential to reduce the stemness property of cancer cells in BC. Taken together, further research (e.g. animal models) are warranted for proof-of-concept of the mechanism as well as to develop miRNA-based treatment strategies in BCSCs. 
